# Dead in the Saddle

**Published:** 1881-06

**Authors:** 


### DEAD IN THE SADDLE.

### THE MYSTERIOUS STRANGER THAT RODE INTO CAMP AT MIDNIGHT.

[Melbourne Correspondence of the London Telegraph.]

I was terribly frightened one night in Queensland by a dead man riding up to my camp-fire at midnight. I was quite alone. I heard my horses neighing and another answering in the Malgas bushes, so I got up and put wood on, making a bright blaze, and presently into the circle of light came a horseman, bending over his pummel, with his large straw hat slouched over his eyes. I took my revolver off my saddle and sang out: "Good night, mate! You travel late. Will you have a drink of tea?" Not a word of answer. Just then my two dogs, who were sniffing about, set up such a terrible cry it made me jump again. After a bit I began to open my eyes to the state of affairs and mustered courage enough to walk up to the horse and take hold of the reins. While doing so I touched the rider's hands, which were as cold as ice, his legs were out of the irons and wound tightly around the mare. I had to cut the reins from the grip of his fingers. I packed him on the horse when sunrise came and led him into Tambo, where I found he was well known as a digger. He had set out thence that morning—after drinking nearly a bottle of brandy—to go to a place distant about forty miles, and I was only twelve miles from the township when he paid his ill-timed visit. There was no doctor within 200 miles at that time. However, they held a kind of inquest, at which the P. M. talked learnedly of muscular contraction and sunstroke, and was puzzled to decide whether the brandy had anything to do with it, as he could swear, from his own experience, that the liquor was first-class. He praised me more than I deserved, for I had half a mind to run away at first. When I am camped out even now alone strange thoughts of that nocturnal horseman come into my head. If any one had told such a story to me I should hardly have credited it—I mean that a man should stick to his horse in that way without any other help than his saddle-straps afforded. His little mare was very quiet though, and was evidently attracted by the sound of my horse-bells.

---

An elderly resident of Newtown was approached by an agent for an encyclopedia. "I guess I won't get one," said the elderly resident, and frankly added, "I know I could never learn to ride one of the pesky things."

